# *CNGC* Gene Family in *Pyrus betulaefolia*: Genome-Wide Analysis and *CNGC4/14* Function in Salt Tolerance via DNA Methylation

**DOI:** 10.3390/ijms27188252

**Published:** 2026-09-16

**Authors:** Hui Li, Jialiang Kan, Yilong Liu, Chunxiao Liu, Xiaogang Li

**Affiliations:** Jiangsu Key Laboratory for Horticultural Crop Genetic Improvement, Institute of Pomology, Jiangsu Academy of Agricultural Sciences, Nanjing 210014, China

**Keywords:** cation transport, *CNGC* gene family, DNA methylation, *Pyrus betulaefolia*, salt stress

## Abstract

Salt stress severely restricts the growth and development of pear trees, and DNA methylation may potentially modulate the salt tolerance of *Pyrus betulaefolia* by regulating ion transporter genes. *Cyclic nucleotide-gated channel* (*CNGC*) family genes are essential for plant ion transport and salt stress adaptation; however, the epigenetic regulatory relationship between DNA methylation and CNGC genes underlying pear salt tolerance remains unclear. In this study, 26 *PbCNGC* genes were systematically identified from *P. betulaefolia*, which possess the conserved motifs characteristic of the CNGC family and can be classified into five subclades. *PbCNGC* members exhibit distinct spatiotemporal expression patterns in ordinary and salt-tolerant genotypes. Under salt stress, core members *PbCNGC3*, *PbCNGC4*, *PbCNGC10*, and *PbCNGC14* displayed typical fluctuating up-and-down expression patterns across roots, stems, and leaves, with distinct spatiotemporal specificity in their expression peaks. In contrast, *PbCNGC19* and *PbCNGC20;1* were exclusively induced in roots under salt stress. Quantitative results showed marked genotypic differences in salt responsiveness. At 4 h of salt treatment, the root transcript levels of *PbCNGC4* and *PbCNGC14* were upregulated by 8.27-fold and 6.76-fold in the salt-tolerant genotype, respectively, which were considerably higher than those in the ordinary genotype (2.08-fold and 4.50-fold). Obvious genotypic differences in mCHH methylation modifications of *PbCNGC4* and *PbCNGC14* were detected after salt treatment, and pharmacological experiments confirmed that DNA methylation negatively modulates the transcription of these two genes. Yeast functional complementation assays further demonstrated that *PbCNGC4* and *PbCNGC14* act as functional Na^+^ and K^+^ permeable cation influx channels. Combined with methylation analysis results, this study reveals a potential regulatory cascade in which DNA methylation may inhibit the transcription of *PbCNGC4* and *PbCNGC14*, modulating their ion-transport capacity, effectively reduces Na^+^ overaccumulation, sustains cellular K^+^ retention to alleviate salt-induced ionic toxicity, and may ultimately shape the salt tolerance of *P. betulaefolia*. These findings enrich the understanding of epigenetic regulation of plant salt tolerance and provide valuable candidate genes and theoretical references for salt-tolerant molecular breeding in pear trees.

## 1. Introduction

Global soil salinization is increasingly aggravated worldwide, posing a major threat to agricultural sustainability and plant growth development [1,2]. Salt stress inhibits plant growth and productivity by disrupting ion homeostasis and inducing osmotic and oxidative damage, where excess reactive oxygen species impair cell membrane stability and photosynthesis [3,4,5]. Plants have evolved multiple adaptive strategies to cope with salinity, including antioxidant defense, phytohormonal signaling, and microbial regulation, with ion transporters central to maintaining Na^+^/K^+^ balance and mitigating salt damage [6,7]. Cyclic nucleotide-gated channels (CNGCs), NHX antiporters, and HAK transporters are core components mediating cation transport and salt signal transduction in plants [7,8,9]. DNA methylation, a key epigenetic modification, modulates the transcription of salt-responsive transporter genes, and genotype-specific methylation patterns contribute to varied salt tolerance by differentially regulating gene expression under salt stress [1,10,11]. Besides endogenous genetic and epigenetic regulation, exogenous strategies effectively improve plant salt resistance through optimizing photosynthetic performance, maintaining intracellular ion homeostasis, and activating stress defense mechanisms to alleviate salt-induced damage [12,13,14,15]. Further investigations of epigenetic regulatory networks, combined with cation transport, will advance our understanding of plant salt adaptation and provide valuable theoretical support and gene resources for salt-tolerant crop molecular breeding.

Pear, as an important temperate fruit crop, is celebrated worldwide owing to its sweet, succulent flesh and wonderful texture [16]. Nevertheless, the continuous degradation of the global ecological environment has led to persistent restrictions on the progression of the pear industry. Consequently, the growth and development of pear trees are inhibited by ionic toxicity and osmotic stress induced by saline conditions [1]. As the original domestication center of pears, China conserves abundant distinctive and elite germplasm resources [2,17]. These germplasm resources consist of experimental materials with diverse genetic backgrounds capable of responding to and mitigating multiple environmental stresses, and carry important practical value for the excavation of pear resistance genes and salt-tolerant breeding research. Among them, *Pyrus betulaefolia* Bunge, an excellent native wild pear species, possesses prominent practical utility. When used as a grafting rootstock, this species can distinctly enhance the salt tolerance of Oriental pears [18].

*P. betulaefolia* employs multiple adaptive strategies to maintain normal growth under salt stress, including restricting Na^+^ translocation to above-ground tissues, regulating Na^+^/K^+^ homeostasis, and enhancing osmotic adjustment [6,10,19]. For instance, specific ion transporters, including *AKT1*, *HAK25*, *NHXs*, and *VHA-B1*, mediate Na^+^ sequestration to maintain cellular ionic and osmotic homeostasis after salt exposure [3,8,10,20]. On the other hand, several genes belonging to the *HAK/KUP/KT* family facilitate the selective absorption of K^+^ and participate in the maintenance of intracellular Na^+^/K^+^ homeostasis [7,21]. Meanwhile, salt stress induces the reprogramming of genomic DNA methylation patterns in the roots of *P. betulaefolia*. Specifically, epigenetic DNA modifications manifested as altered CHH methylation levels modulate the transcription of Ca^2+^ sensor genes *PbCDPK20.1/20.2* as well as their downstream functional genes *PbAKT1* and *PbHAK25*, which further stabilizes Na^+^/K^+^ homeostasis in root tissues [10]. Nevertheless, it is still unclear whether this DNA methylation regulatory cascade acts on other ion transport genes. Indeed, elucidating the correlation between dynamic methylation variations and the transcriptional abundance of different ion transporter genes upon salt stress is indispensable to completely decipher the molecular framework whereby DNA methylation governs salt tolerance in *P. betulaefolia*.

Plant cyclic nucleotide-gated channels (CNGCs) are evolutionarily conserved non-selective cation channels (permeable to Na^+^, K^+^, Ca^2+^) that regulate Na^+^ homeostasis through specific signaling pathways, thus participating in salt tolerance regulation [9]. CNGCs primarily function as direct mediators of ion transport. In *Arabidopsis*, *AtCNGC10* promotes root Na^+^ influx. Suppression of its expression reduces Na^+^ accumulation and enhances salt tolerance [22]. Conversely, its antisense lines exhibit higher shoot Na^+^ content than the wild-type line [23]. *AtCNGC4* can be activated by cyclic nucleotides and exhibits equal permeability to Na^+^ and K^+^ [24]. But *AtCNGC19* and *AtCNGC20* function as functionally redundant genes, which single-gene mutations fail to alter Na^+^ accumulation and salt tolerance phenotypes in plants [25]. Previous studies also revealed similar roles for *CNGCs* in other plant species. Kenaf *HcCNGC21* enhances salt tolerance through Na^+^ transport [26], whereas soybean *GsCNGC20-d* is involved in Ca^2+^ efflux and Na^+^/K^+^ uptake [27]. Besides directly transporting ions, *CNGCs* still participate in maintaining plant ion homeostasis. For example, certain root-expressed *CNGC* members rapidly activate the Na^+^/K^+^ homeostasis pathway. *AtCNGC10* mediates root cation uptake and shoot ion allocation [23]; its homolog *AtCNGC3* exhibits similar functions [28]. *GsCNGC20-d* confers salt tolerance in soybean by maintaining a low shoot Na^+^/K^+^ ratio [29]. Notably, CNGC expression is dynamically regulated by salt stress signals and further integrates with multiple signaling pathways to form a molecular regulatory network [29,30,31,32,33]. In addition, demethylation of certain *CNGC* genes not only serves as a prerequisite for their transcriptional activation but also acts as a key initiating node within the stress-response network [34]. Taken together, the above results establish a theoretical basis for dissecting the regulatory functions of *CNGC* family genes underlying salt tolerance in *P. betulaefolia*.

This study aims to fill the knowledge gap regarding the epigenetic regulation of the *cyclic nucleotide-gated channel* (*CNGC*) gene family underlying salt tolerance in *P*. *betulaefolia*. Previous studies have demonstrated that the salt-tolerant *P. betulaefolia* genotype copes with salt stress by maintaining a higher root K^+^/Na^+^ ratio and reducing Na^+^ accumulation in aerial tissues. Furthermore, the expression of potassium transporter genes (e.g., *PbAKT1* and *PbHAK25*) is negatively regulated by their promoter methylation levels [10]. Nevertheless, whether the *CNGC* gene family participates in salt stress responses through a similar methylation regulatory mechanism remains unclear. Accordingly, the present study established the following specific objectives. First, we comprehensively identify the members of the *CNGC* gene family in the *P. betulaefolia* genome and analyze their sequence characteristics, evolutionary relationships, as well as their spatiotemporal expression profiles in both ordinary and salt-tolerant genotypes. Second, yeast complementation assays are performed to functionally verify the Na^+^ and K^+^ transport capacities of candidate *CNGC* genes, particularly *PbCNGC4* and *PbCNGC14*. Third, pharmacological treatments are applied to manipulate the methylation level of *CNGC* genes, and the resulting transcriptional changes are detected. This study intends to clarify whether DNA methylation improves salt tolerance in *P. betulaefolia* by modulating the expression of *CNGC* genes, thereby providing experimental evidence for elucidating the comprehensive role of DNA methylation in coordinating salt tolerance mechanisms in pear rootstock.

## 2. Results

### 2.1. PbCNGCs Identification and Bioinformatics Analysis

A total of 26 *CNGC* genes were screened and identified from the *P. betulaefolia* genome. Domain analysis revealed that each candidate gene encodes a cyclic nucleotide monophosphates binding domain (CNBD), six transmembrane domains with a pore loop (P-loop), and at least one calmodulin binding domain (CaMBD) (Table 1). These genes were systematically named according to the sequence homology between *PbCNGCs* of *P. betulaefolia* and CNGC family members from *P. bretschneideri*, the detailed information of which was summarized in Table 1. Among these *PbCNGC* genes, all but three (namely *PbCNGC11;1*, *PbCNGC13*, and *PbCNGC21*) were found to possess a distinct isoleucine-glutamine (IQ) calmodulin-binding motif. Further characterization of their physicochemical properties showed that their corresponding CDS regions range from 1782 to 2424 bp in length. The deduced amino acid sequences of these proteins contain 593 to 807 residues, with calculated molecular weights ranging from 68.78 kDa to 91.73 kDa. Isoelectric point analysis indicated that all PbCNGC proteins have pI values between 8.35 and 10.05, which are above 7, classifying them as alkaline proteins. Furthermore, subcellular localization prediction suggested that all identified PbCNGC proteins are targeted to the plasma membrane.

**Figure 1:**
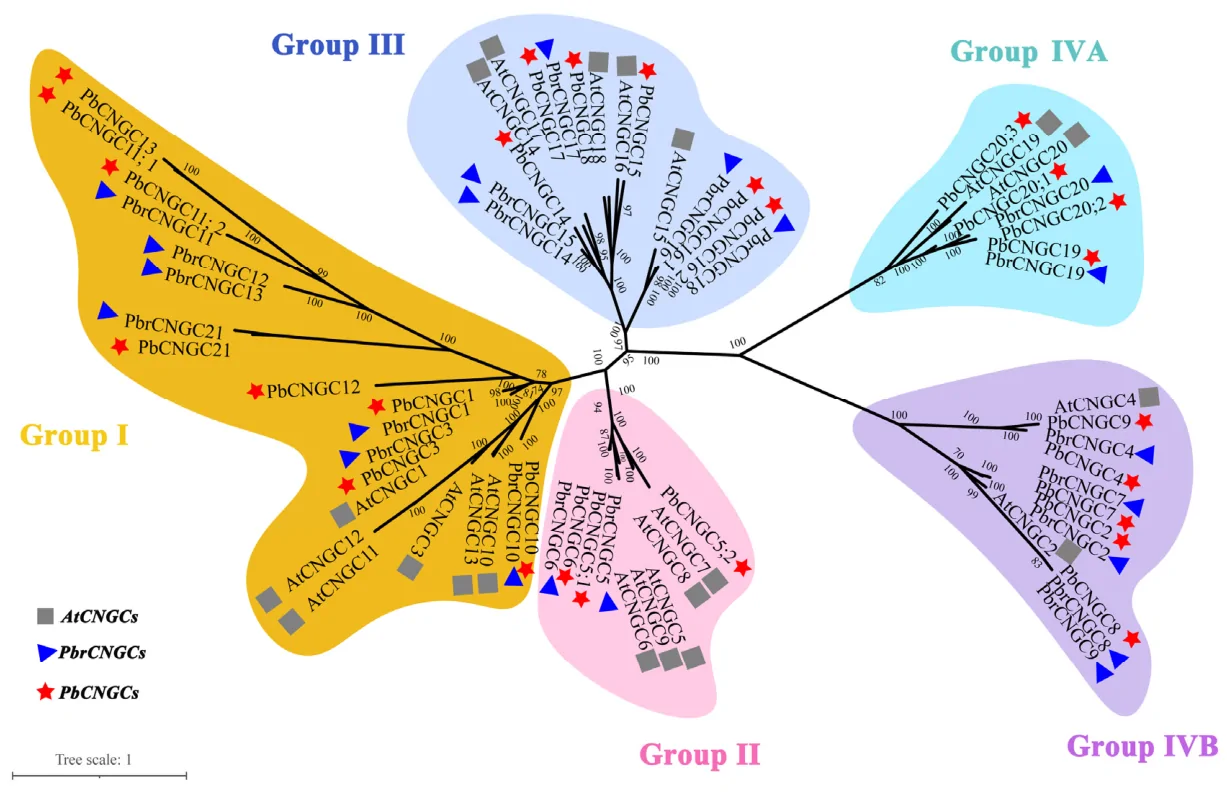
Phylogenetic relationship among cyclic nucleotide-gated channels (CNGCs) from *Pyrus betulaefolia*, *Pyrus bretschneideri*, and *Arabidopsis*. The preliminary nomenclature of these *P. betulaefolia* CNGCs was assigned to be consistent with that of *P. bretschneideri* CNGCs. Neighbor-joining phylogenetic tree shows the relationship among 26 *P. betulaefolia*, 21 *P. bretschneideri*, and 20 *Arabidopsis* CNGC proteins. The resulting five group names are labeled as I, II, III, IV-A, IV-B. Tree visualization and optimization were implemented on EvolView (https://www.evolgenius.info/evolview/, accessed on 11 March 2025), and the scale bar represents a genetic distance of 1.0.

To investigate the classification and evolutionary relationships of the *P. betulaefolia PbCNGC* genes, a phylogenetic analysis was performed based on their deduced amino acid sequences. A maximum likelihood (ML) phylogenetic tree revealed that all *PbCNGC* genes could be divided into four highly supported clades, with bootstrap values ranging from 95% to 100% (Figure 1). Notably, the previously designated clade IV was further split into two distinct subclades. Collectively, the 26 *PbCNGC* genes were clustered into five groups, namely Groups I, II, III, IVA, and IVB (Figure 1). Group I represented the largest clade, comprising eight members, while the remaining groups contained three to six members each. Furthermore, although the *CNGC* genes of pear and *Arabidopsis* formed a monophyletic cluster within Group I, the substantial genetic distance between them suggested that these genes might have undergone independent amplification events. The number of *CNGC* members in Group III was approximately equivalent between pear and *Arabidopsis*. In contrast, pronounced disparities in member numbers were observed in Groups II and IVB: the *PbCNGC* family contained more members in Group IVB (five members) but fewer in Group II (three members). Taken together, these findings suggest that frequent gene duplication events occurred in the *PbCNGC* subfamilies after the divergence of pear from other species, thereby contributing to the rapid expansion of the *PbCNGC* gene family in *P. betulaefolia*.

**Figure 2:**
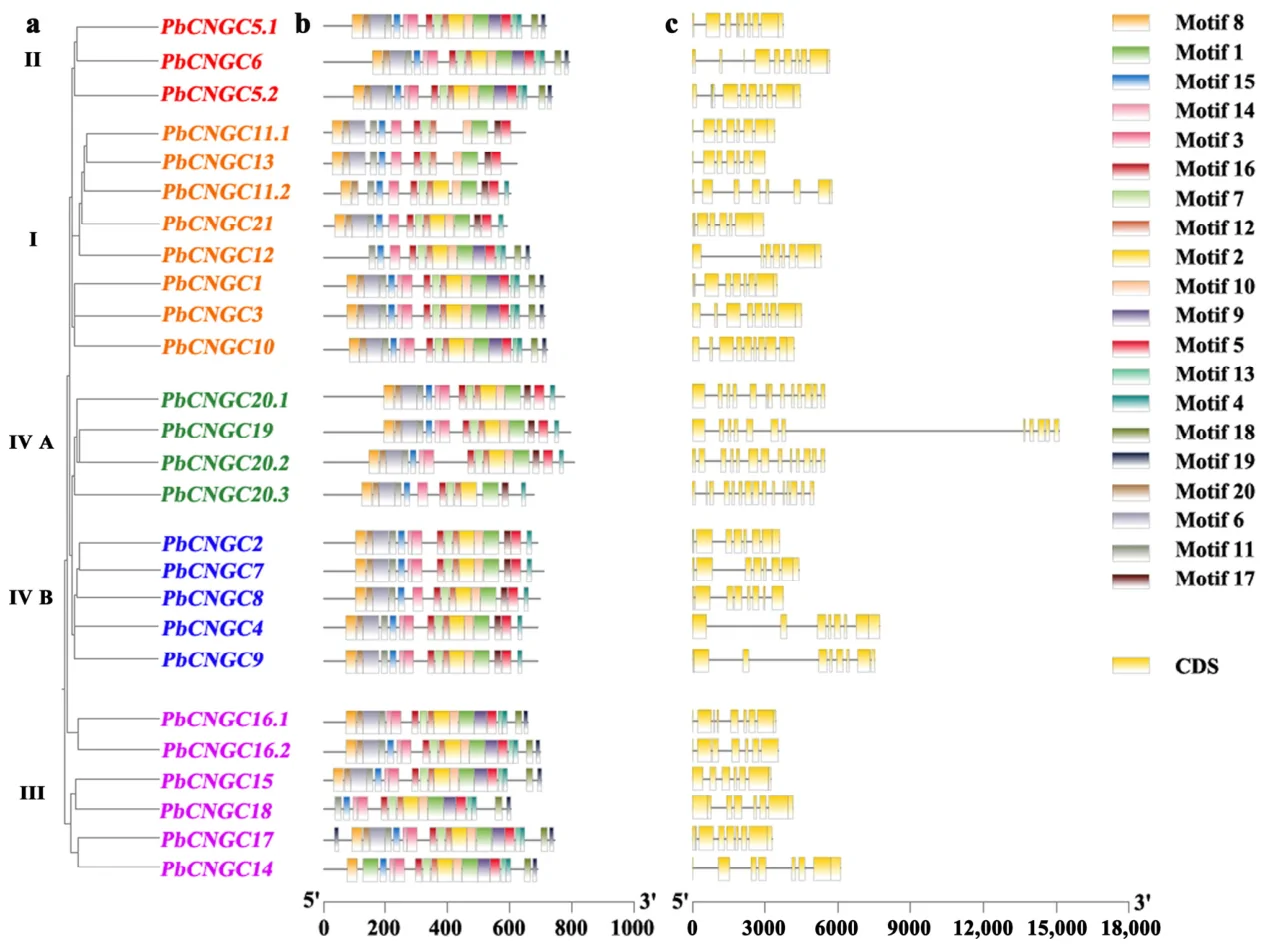
The phylogenetic tree, motifs, and gene structure analysis of *PbCNGCs.* (**a**) Phylogenetic tree of *PbCNGCs*; (**b**) motifs of *PbCNGCs*; (**c**) gene structures of *PbCNGCs*.

**Figure 3:**
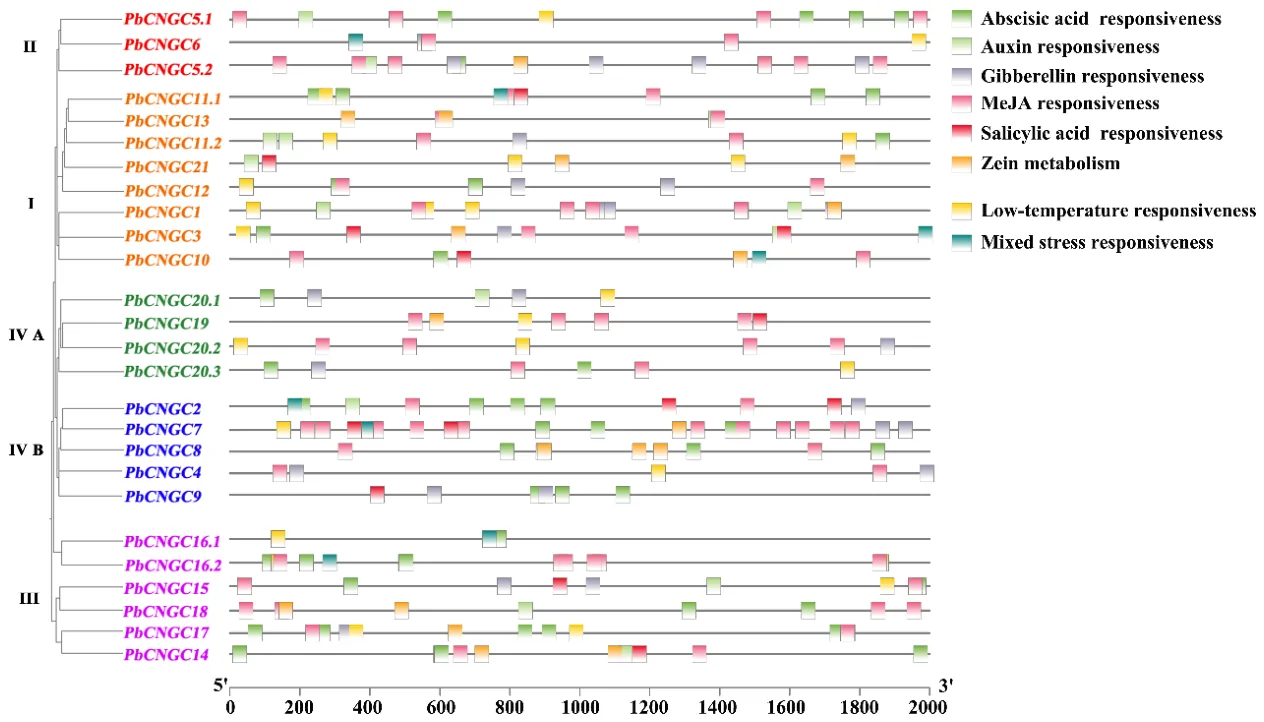
The phylogenetic tree of *PbCNGCs* from *Pyrus betulaefolia* was constructed using the neighbor-joining (NJ) method with 1000 replicates of bootstrap validation. The types, numbers, and locations of cis-elements in promoter regions 2 kb upstream of *PbCNGC* genes were checked by using the PlantCARE database (https://bioinformatics.psb.ugent.be/webtools/plantcare/html/, accessed on 12 March 2025).

Motif analysis revealed that pear CNGCs contain a diverse set of motifs (Figure 2) and exhibit high similarity in both motif quantity and distribution, particularly among *PbCNGCs* belonging to the same subfamily. Among the 26 PbCNGC proteins, PbCNGC17 contained 20 motifs, while the other members possessed 13 to 19 motifs (Figure 2). Gene structure analysis of *PbCNGCs* showed that the number of CDSs per gene ranged from 6 to 15. Specifically, *PbCNGC20;1*, *PbCNGC20;2*, and *PbCNGC20;3* each contained more than ten exons (Figure 2). Cis-element analysis demonstrated that every *PbCNGC* family member harbors plant hormone-responsive motifs, and low-temperature responsive cis-elements are detected in the majority of these genes (Figure 3). Furthermore, eight members (*PbCNGC2*, *3*, *6*, *7*, *10*, *11;1*, *16;1*, and *16;2*) possess mixed stress response elements (Figure 3).

**Figure 4:**
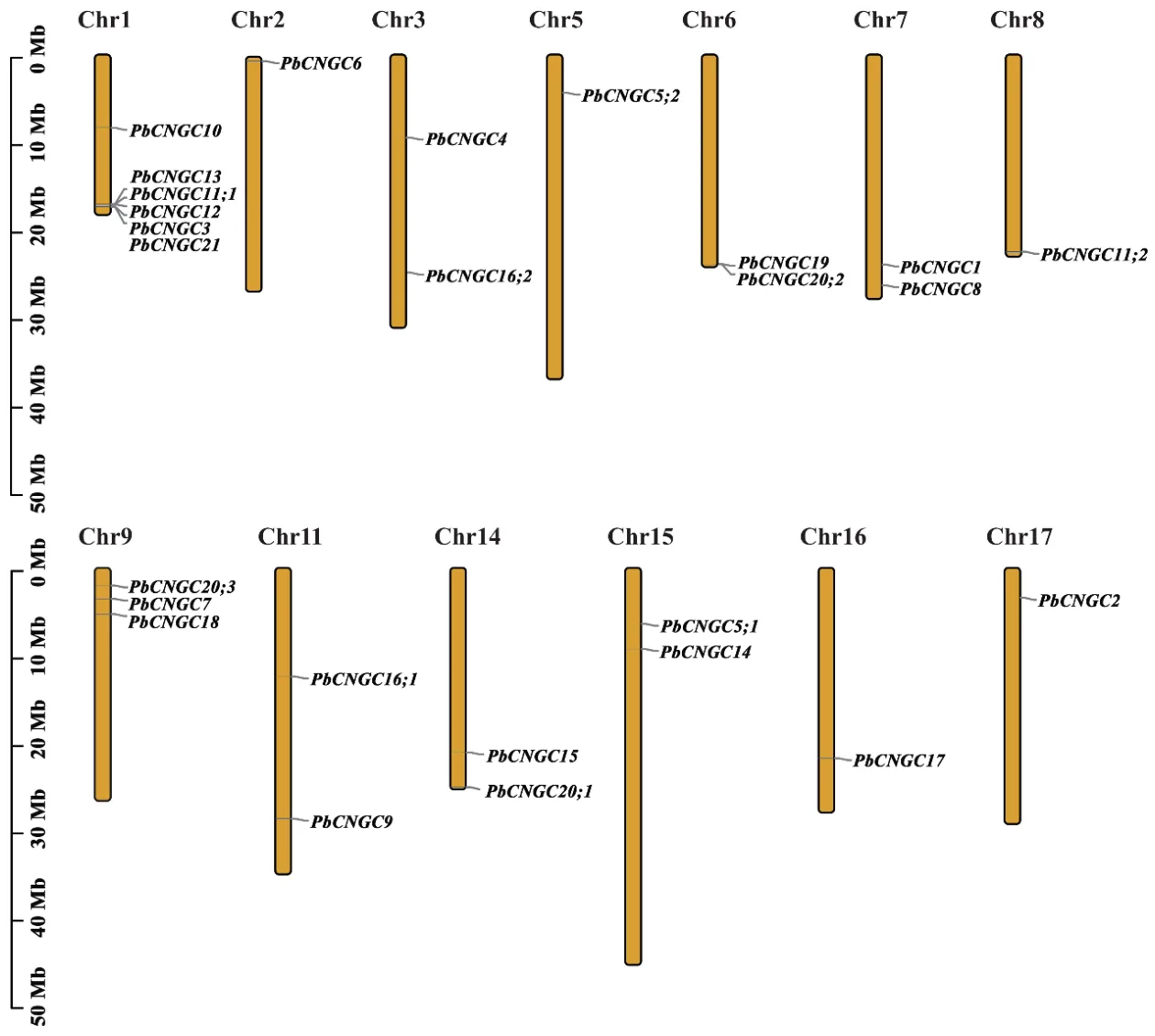
Distributions of *PbCNGC* genes on the *Pyrus betulaefolia* chromosomes. The scale is shown on the left.

Based on the genomic annotation data of *P. betulaefolia*, the chromosomal distribution of *PbCNGC* genes was mapped (Figure 4). The 26 *PbCNGC* genes were unevenly distributed across 17 pear chromosomes. Chromosome 1 harbored the largest number of *PbCNGC* genes, with a total of six members. Chromosome 9 carried three *PbCNGC* genes, while chromosomes 3, 6, 7, 11, 14, and 15 each contained two members. Only one *PbCNGC* gene was detected on each of chromosomes 2, 5, 8, 16, and 17.

### 2.2. Response of PbCNGC Genes to Salt Stress

**Figure 5:**
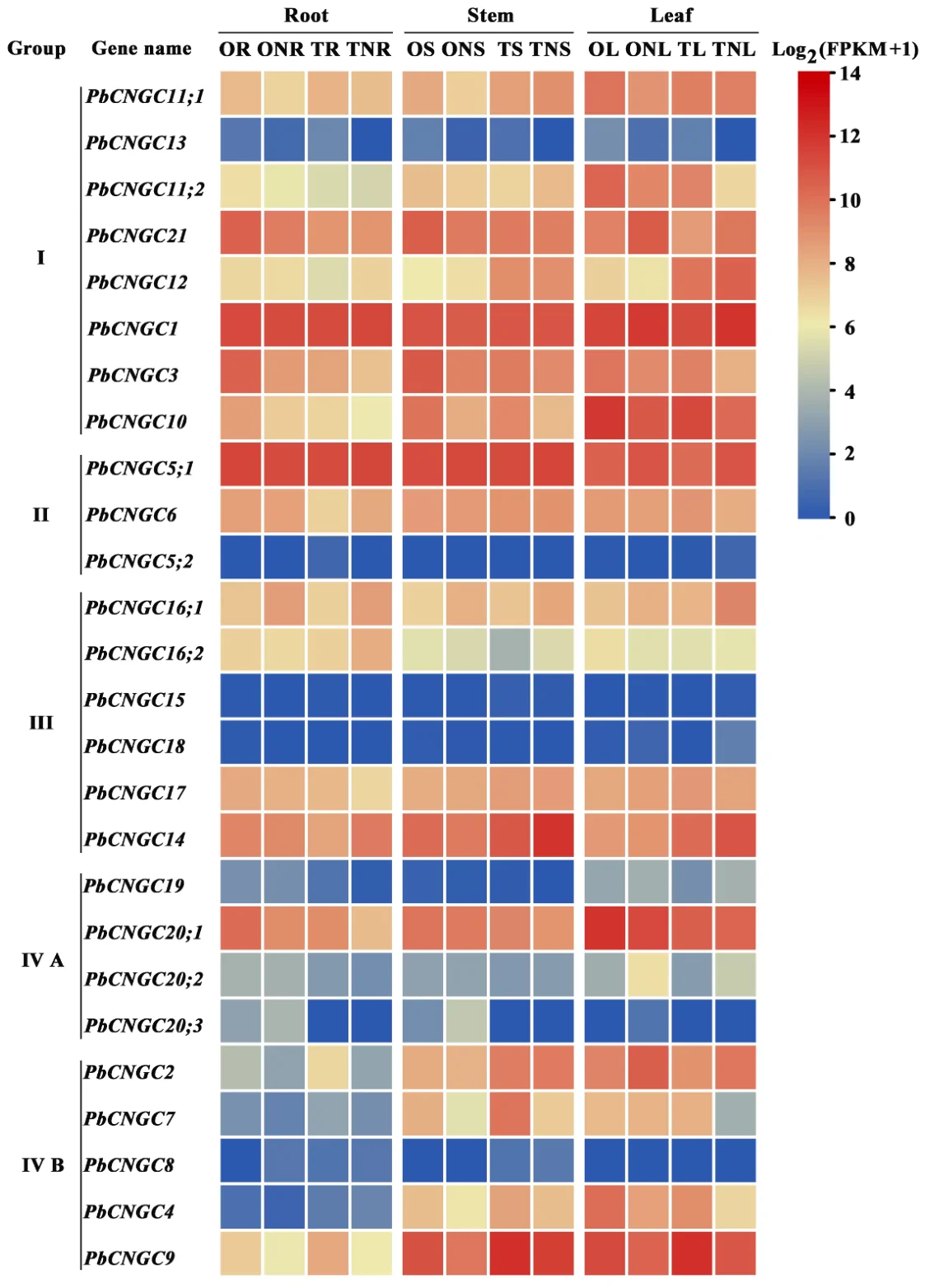
The expression patterns of *PbCNGCs* in two ecotypes of *Pyrus betulaefolia* under salt stress. OR, OS, and OL represent the roots, stems, and leaves of the ordinary genotype under normal conditions, respectively. ONR, ONS, and ONL represent the roots, stems, and leaves of the ordinary genotype under salt stress, respectively. TR, TS, and TL represent the roots, stems, and leaves of the salt-tolerant genotype under normal conditions, respectively. TNR, TNS, and TNL represent the roots, stems, and leaves of the salt-tolerant genotype under salt stress, respectively.

**Figure 6:**
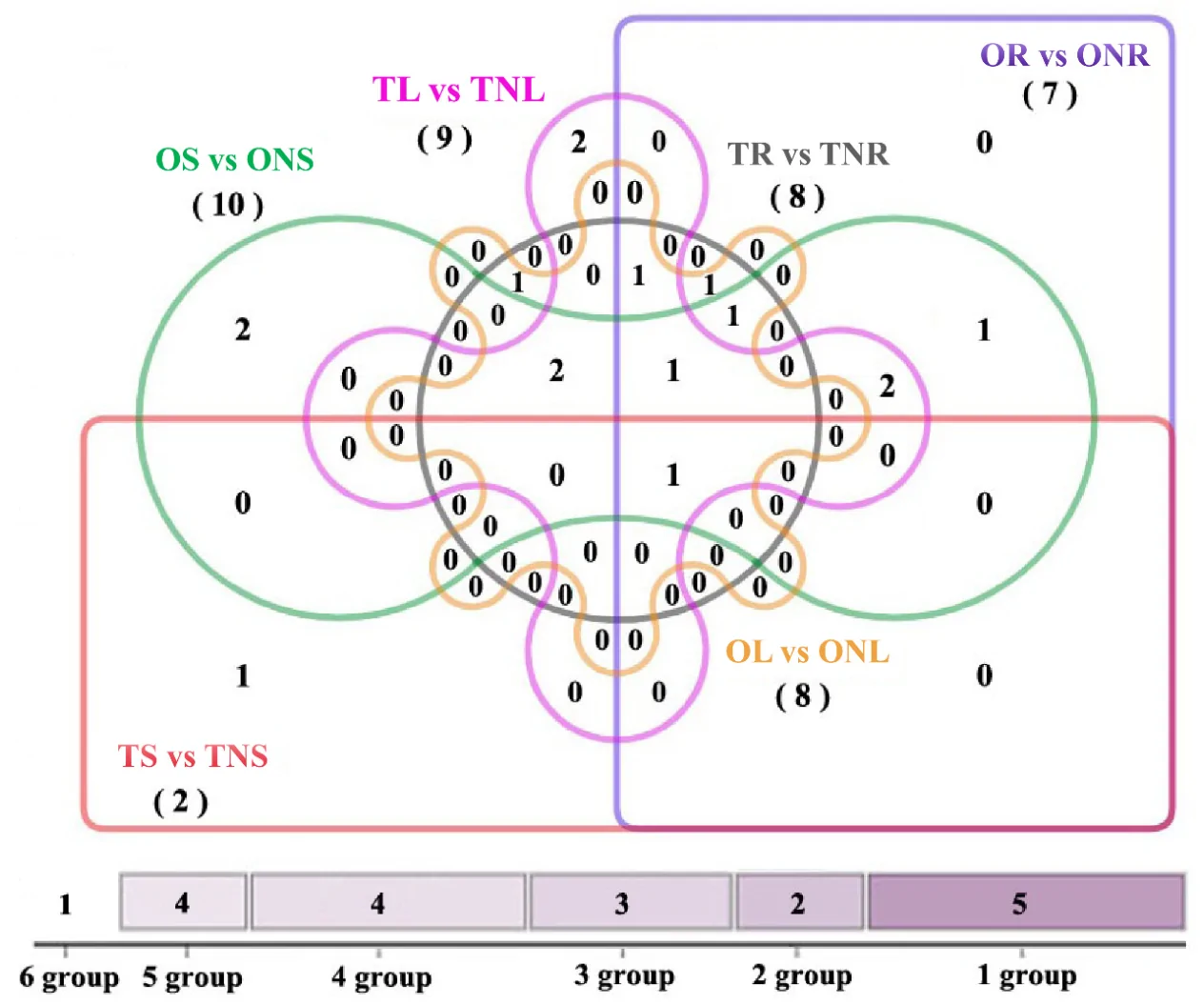
Venn diagram of *PbCNGCs* which were differentially expressed in two ecotypes of *Pyrus betulaefolia* after salt stress. OR, OS, and OL represent the roots, stems, and leaves of the ordinary genotype under normal conditions, respectively. ONR, ONS, and ONL represent the roots, stems, and leaves of the ordinary genotype under salt stress, respectively. TR, TS, and TL represent the roots, stems, and leaves of the salt-tolerant genotype under normal conditions, respectively. TNR, TNS, and TNL represent the roots, stems, and leaves of the salt-tolerant genotype under salt stress, respectively.

To characterize the biological functions of the 26 *PbCNGC* genes in response to salt stress, transcriptome data (NCBI, project number PRJNA812627) from ordinary genotype (O) and salt-tolerant genotype (T) of *P. betulaefolia* were used to profile gene expression across root, stem, and leaf tissues. Results showed that most *PbCNGCs* exhibited transcriptional signals in the three tested tissues, whereas their primary expression tissues varied substantially across individual genes (Figure 5). Prior to salt stress treatment, *PbCNGC2*, *4*, *7*, and *9* accumulated abundant transcripts in both leaves and stems; by contrast, *PbCNGC10*, *PbCNGC11;2*, and *PbCNGC20;1* were preferentially expressed solely within leaf tissue. After 24 h of 200 mM NaCl treatment, 12 genes displayed identical expression patterns in the ordinary genotype (O) and salt-tolerant genotype (T). Within this subset, *PbCNGC2/3/9/20;1*, *PbCNGC7*, and *PbCNGC4* experienced transcriptional down-regulation in roots, stems, and stems plus leaves, respectively. Meanwhile, the transcript abundance of *PbCNGC10* and *PbCNGC13* was suppressed in all three tested organs (roots, stems, and leaves). In contrast, transcription of *PbCNGC18*, *PbCNGC20;2*, and *PbCNGC21* in leaves was up-regulated upon salt stress, and *PbCNGC16;1* showed induced expression across roots, stems, and leaves. The rest of the *PbCNGC* family members displayed differential transcriptional remodelling in multiple organs when comparing the two genotypes (Figure 5 and Figure 6). All results indicated that *PbCNGC* genes perform their primary biological functions in different tissues.

**Figure 7:**
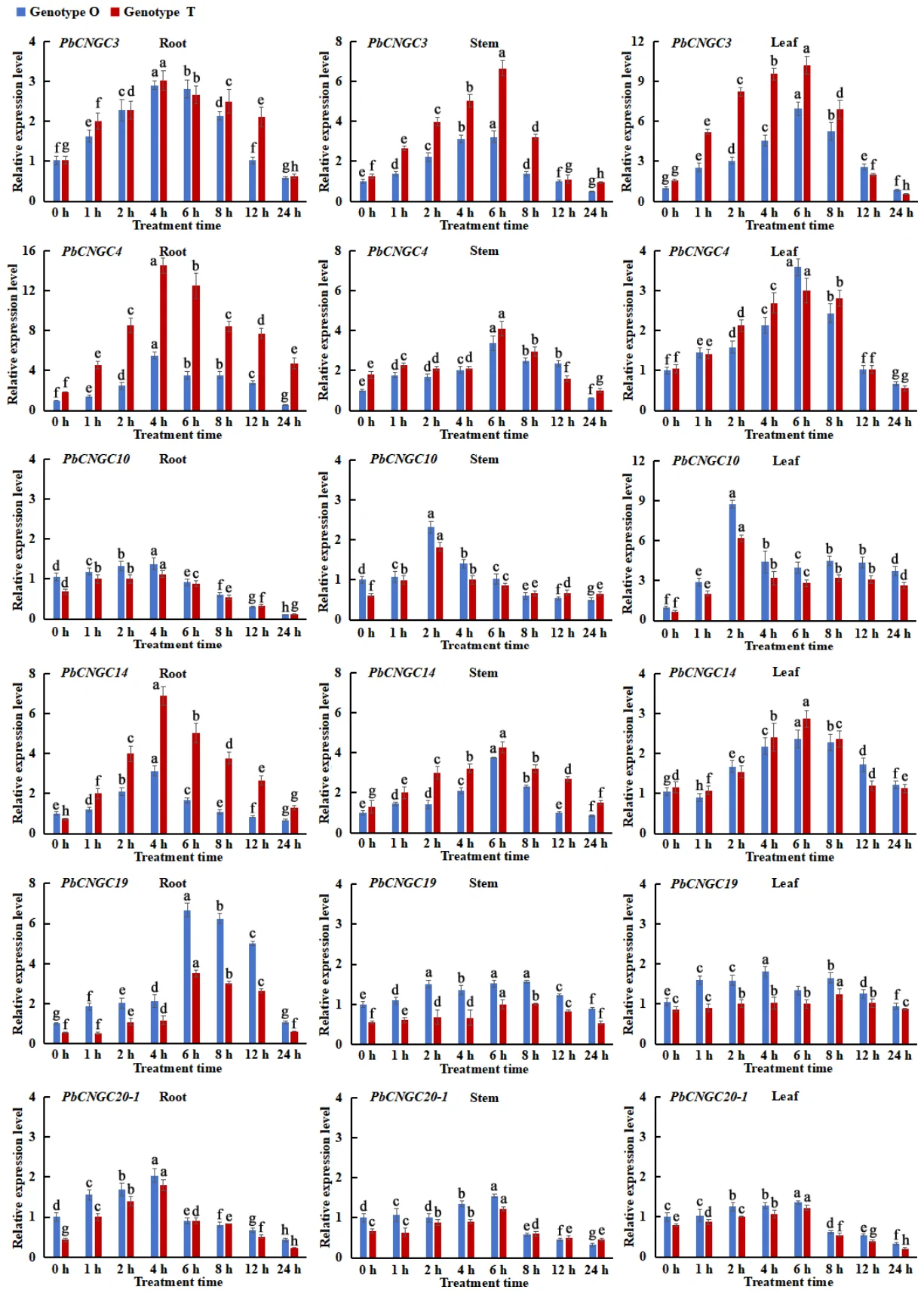
Expression status of *PbCNGCs* in roots, stems, and leaves of two ecotypes from *Pyrus betulaefolia* after salt stress. Values are presented as means ± standard deviations (SDs). Vertical bars indicate SDs of the means from no fewer than three biological replicates. Values marked with different superscript letters differ significantly among treatments (*p* < 0.05; one-way analysis of variance followed by Tukey’s Honestly Significant Difference test).

To further characterize the expression dynamics of *PbCNGC* genes under salt stress, qPCR was performed to quantify their transcript levels in six family members (Figure 7). The transcript levels of the six *PbCNGC* genes were initially induced and subsequently repressed under prolonged salt stress, indicating their involvement in plant salt response regulation. Under salt stress, *PbCNGC3*, *PbCNGC4*, *PbCNGC10*, and *PbCNGC14* exhibited a typical dynamic up-and-down regulation pattern in roots, stems, and leaves, with distinct temporal and spatial specificity in their peak expression. In roots, *PbCNGC3*, *PbCNGC4*, *PbCNGC10*, *PbCNGC14*, and *PbCNGC20;1* reached peak transcript abundance at 4 h of salt treatment, whereas *PbCNGC19* peaked later at 6 h. In stems and leaves, *PbCNGC3*, *PbCNGC4*, and *PbCNGC14* achieved their maximum transcript levels at 6 h, while *PbCNGC10* displayed an earlier response and peaked at 2 h. In contrast, *PbCNGC19* and *PbCNGC20;1* showed strict root-specific salt responsiveness; their expression was significantly induced only in roots and remained stable without obvious temporal fluctuations in stems and leaves. In terms of tissue-dependent expression magnitude, *PbCNGC4* and *PbCNGC14* exhibited substantially higher fold changes in roots than in stems and leaves under salt stress, while *PbCNGC3* and *PbCNGC10* showed stronger upregulation in leaves than in other tissues. Moreover, the induction extent differed significantly between the two genotypes. At 4 h of salt treatment, the fold changes of *PbCNGC4* and *PbCNGC14* in the roots of the salt-tolerant genotype reached 8.27-fold and 6.76-fold, respectively, which were markedly higher than those in the ordinary genotype (2.08-fold and 4.50-fold).

### 2.3. DNA Methylation Changes Influence PbCNGC4/14 Expression Under Salt Stress

**Figure 8:**
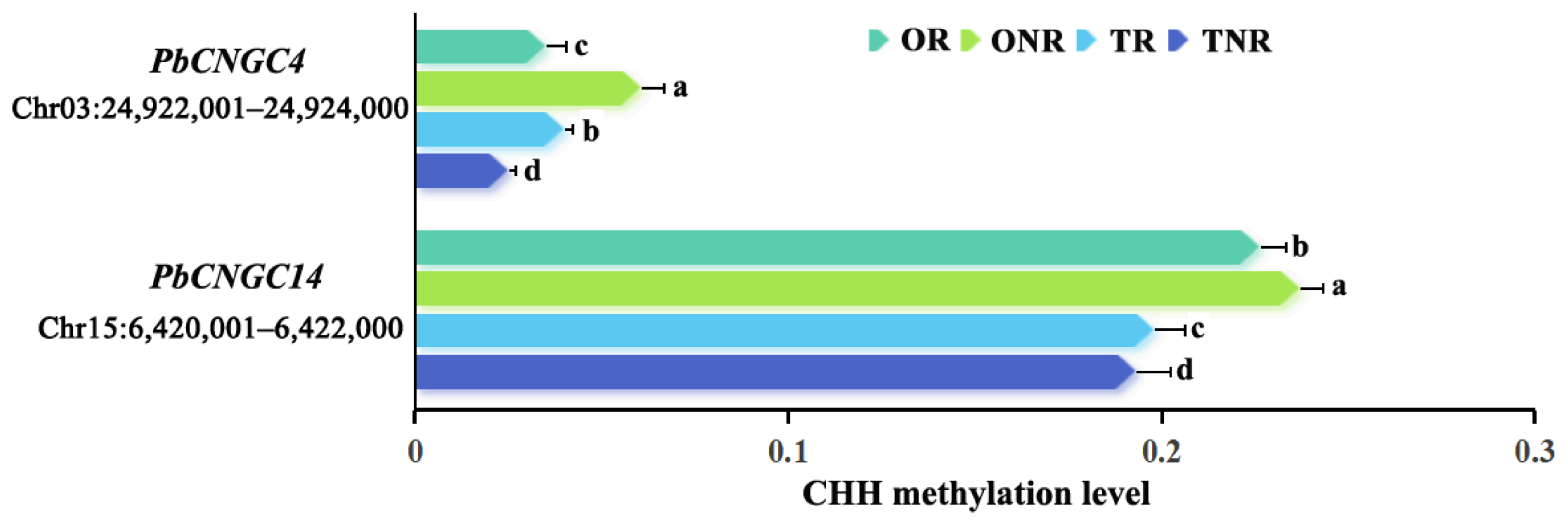
Methylation level in the reading windows of differentially methylated *PbCNGC4* and *PbCNGC14* genes in roots of *Pyrus betulaefolia* after salt stress. OR, roots of the ordinary genotype under normal conditions; ONR, roots of the ordinary genotype under salt-stress conditions; TR, roots of the salt-tolerant genotype under normal conditions; TNR, roots of the salt-tolerant genotype under salt-stress conditions. Values marked with different superscript letters differ significantly among treatments (*p* < 0.05; one-way analysis of variance followed by Tukey’s Honestly Significant Difference test).

To explore whether salt-responsive transcriptional variation is modulated by dynamic DNA methylation, we used MethylKit (v1.12.0, span = 1000) [10] to screen root differentially methylated regions (DMRs) in two *P. betulaefolia* genotypes under salt treatment and control conditions. DMR functional annotation was performed via Bedtools (v2.31.0). Then, two DMRs were matched to the genomic intervals of *PbCNGC4* and *PbCNGC14* in birch-leaf pear roots. More concretely, upon 24 h of 200 mM NaCl treatment, the salt-tolerant genotype exhibited reduced methylation levels within the *PbCNGC4* locus (Chr03:24922001–24924000) and the *PbCNGC14* genomic interval (Chr15:6420001–6422000) (Figure 8). In contrast, these two regions exhibited increased methylation levels in the ordinary genotype under the same condition (Figure 8). In addition, the differentially methylated regions within the gene sequences of *PbCNGC4* and *PbCNGC14* belonged to the mCHH methylation type. Specifically, the differential methylation region of *PbCNGC4* was located in the intron region, while that of *PbCNGC14* was situated in the promoter region (Table 2). Collectively, these two genes exhibited distinct methylation patterns between the salt-tolerant and ordinary genotypes.

**Figure 9:**
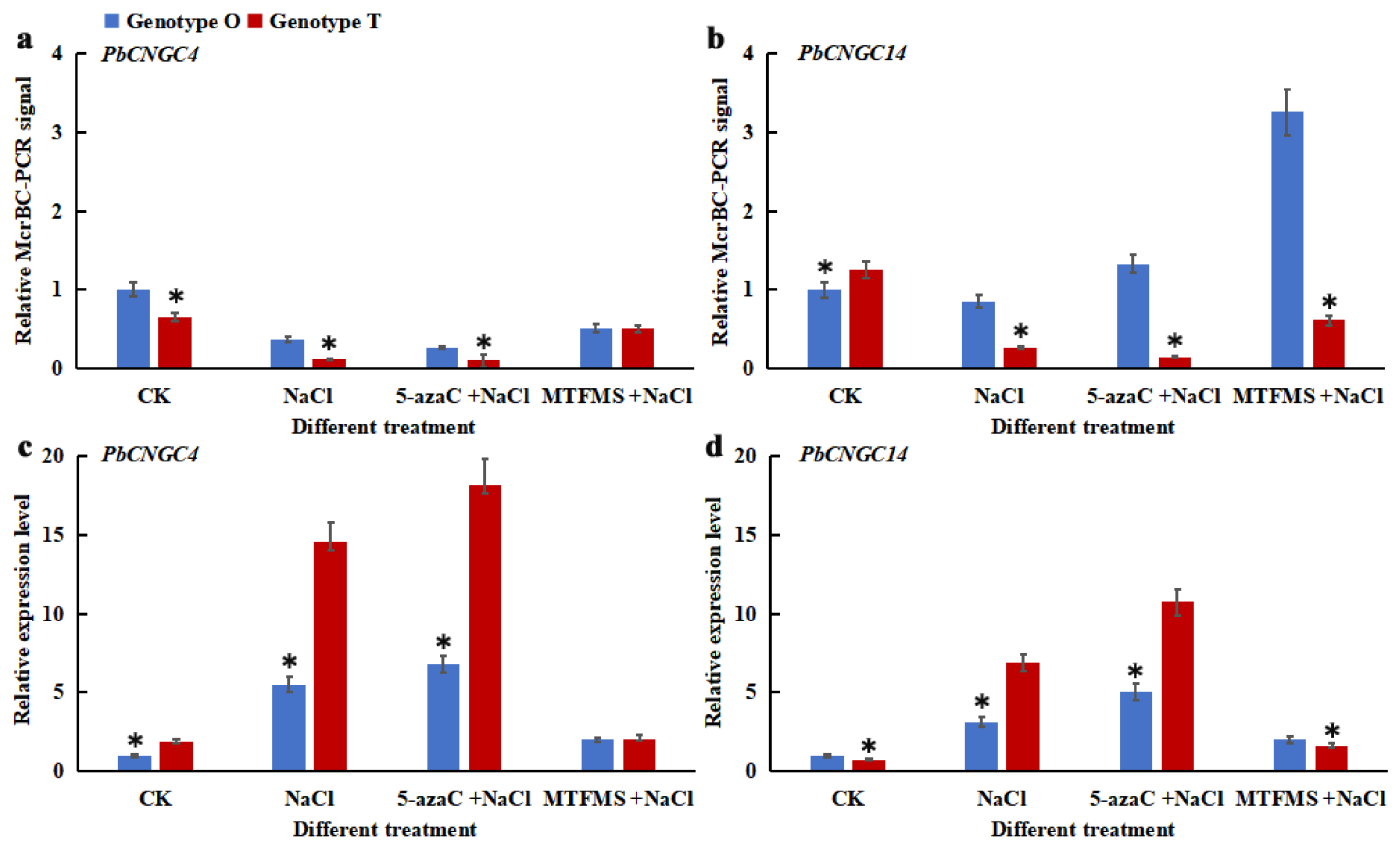
Effects of the DNA methyltransferase inhibitor 5-azacytidine (5-azaC) and accelerator methyl trifluoromethanesulfonate (MTFMS) on DNA methylation and transcript levels of *PbCNGC4* and *PbCNGC14* in two ecotypes of *Pyrus betulaefolia* under salt-stress conditions. (**a**) Relative McrBC-PCR signal of *PbCNGC4*. (**b**) Relative McrBC-PCR signal of *PbCNGC14*. (**c**) Relative expression level of *PbCNGC4*. (**d**) Relative expression level of *PbCNGC14*. Methylated DNA can be digested by McrBC, and thus, higher qPCR signals indicate lower methylation levels (MLs). Values are presented as the means ± standard deviations (SDs). Vertical bars represent the SDs of the means from at least three biological replicates, and values with asterisks (*) are considered significantly different between the two *P. betulaefolia* genotypes (*p* < 0.05, Hypothesis Testing followed by Two-Sample *t*-Test, performed using Origin 2025).

To verify whether DNA methylation regulates the expression of *PbCNGC4* and *PbCNGC14* in *P. betulaefolia* roots, we treated the ordinary genotype (O) and salt-tolerant genotype (T) with the DNA methylation inhibitor 5-azaC and methylation accelerator MTFMS for 12 h, followed by 200 mM NaCl salt-stress treatment for 4 h. Intrinsic genotypic differences in DNA methylation existed between the two genotypes. Under control conditions, salt-tolerant genotype T displayed significantly lower methylation levels and higher transcript abundance of *PbCNGC4* and *PbCNGC14* relative to ordinary genotype O. Upon NaCl exposure, methylation levels of both genes declined in both genotypes, yet genotype T still preserved hypomethylation and higher gene expression (Figure 9a–d). Pharmacological manipulation supported our expectations. Under salt stress, 5-azaC decreased DNA methylation and significantly up-regulated *PbCNGC4* and *PbCNGC14* transcription in both genotypes, and diminished genotypic divergence in methylation and gene expression in genotype O. By contrast, MTFMS elevated methylation and repressed gene transcription; it notably strengthened *PbCNGC14* methylation and suppressed its expression in genotype O, while genotype T retained relatively low methylation. The transcriptional changes of *PbCNGC4* and *PbCNGC14* were tightly correlated with DNA methylation dynamics upon 5-azaC and MTFMS treatments: 5-azaC reduced target-gene methylation to elevate their expression under salt stress, whereas MTFMS caused opposite changes in methylation and transcript abundance. Collectively, these results confirm that DNA methylation critically modulates *PbCNGC4* and *PbCNGC14* transcription. Higher methylation confers stronger transcriptional repression, whereas hypomethylation facilitates transcript accumulation. Thus, genotype-specific basal DNA methylation differences govern the transcriptional activation of *PbCNGC4* and *PbCNGC14* under salt stress. This may partly explain the increased Na^+^ storage and higher K^+^/Na^+^ ratio observed in roots of the salt-tolerant ecotype [10].

### 2.4. PbCNGC4/14 Complements Yeast Mutants Defective in Na^+^/K^+^ Uptake

**Figure 10:**
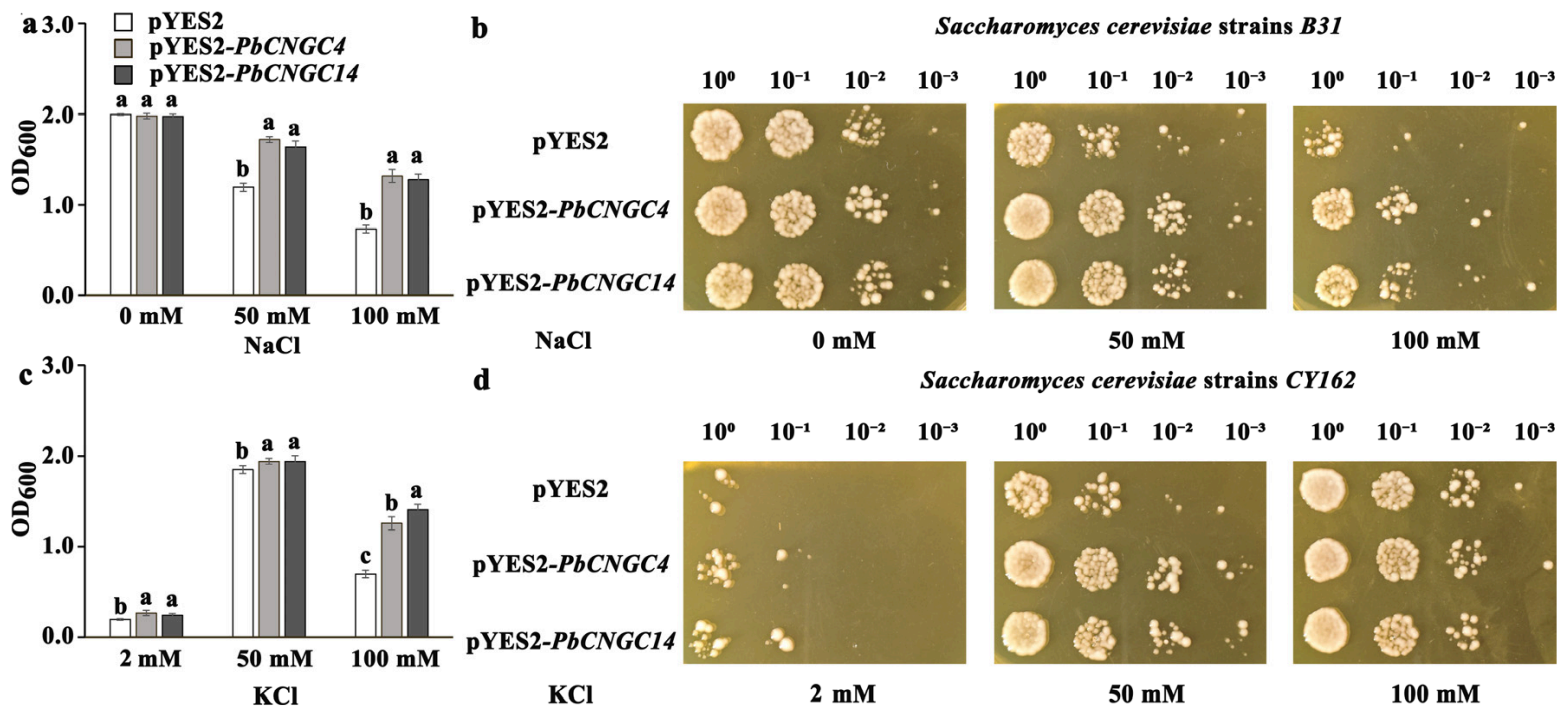
Complementation of Na^+^ and K^+^ transport mutants of *Saccharomyces cerevisiae* by *PbCNGC4 and PbCNGC14* from *Pyrus betulaefolia.* (**a**) Complementation of the *B31* yeast strain on AP medium containing 0, 50, or 100 mM NaCl. *B31* was transformed with plasmids containing pYES2-*PbCNGC4*, pYES2-*PbCNGC14*, or an empty vector pYES2 (negative control). Photographs were taken after incubation at 30 °C for 72 h. (**b**) The OD_600_ values of recombinant yeast after 48 h of cultivation in AP liquid medium supplemented with 0, 50, or 100 mM NaCl. (**c**) Complementation of the *CY162* yeast strain on potassium-free modified YNB medium containing 2, 50, or 100 mM KCl. *CY162* was transformed with plasmids containing pYES2-*PbCNGC4*, pYES2-*PbCNGC14*, or an empty vector pYES2 (negative control). Photographs were taken after incubation at 30 °C for 72 h. (**d**) The OD_600_ values of recombinant yeast after 48 h of cultivation in potassium-free modified YNB medium supplemented with 2, 50, or 100 mM KCl. Values are means ± standard error (SE, *n* = 3). Different lowercase letters indicate significant differences (*p* < 0.05) among transformed yeast strains, based on one-way analysis of variance followed by Tukey’s Honestly Significant Difference test, performed using Origin 2025.

To verify whether *PbCNGC4* and *PbCNGC14* confer cation channel activity and mediate transmembrane ion transport, we performed heterologous expression of these two genes in yeast mutants with impaired cation transport capacity. The yeast strain *B31* lacks the key Na^+^-efflux ATPase genes ENA1-4, leading to drastically increased salt sensitivity [22]. Under NaCl-free AP medium, yeast cells transformed with the empty vector pYES2 or recombinant vectors pYES2-*PbCNGC4/PbCNGC14* exhibited consistent growth performance. However, distinct growth differences were observed among the three groups after 72 h of cultivation on solid medium and 48 h of incubation in liquid medium supplemented with 50 mM or 100 mM NaCl (Figure 10a,b). Compared with the severely growth-inhibited pYES2 control cells, yeast strains expressing *PbCNGC4* or *PbCNGC14* showed substantially enhanced salt tolerance (Figure 10a,b). The yeast strain *CY162* lacks the high-affinity K^+^ uptake system Trk1/Trk2/Tok1, which impairs cellular potassium absorption [35]. All yeast strains failed to grow normally on YNB solid and liquid medium containing only 2 mM KCl, whereas the addition of 50 mM or 100 mM KCl partially restored their growth defects (Figure 10c,d). Notably, heterologous expression of *PbCNGC4* and *PbCNGC14* obviously promoted yeast growth relative to the pYES2 empty vector control (Figure 10c,d). Collectively, these results demonstrate that *PbCNGC4* and *PbCNGC14* act as Na^+^- and K^+^-permeable cation influx channels in yeast and may participate in regulating Na^+^/K^+^ homeostasis during salt-stress adaptation in *P. betulaefolia*.

## 3. Discussion

### 3.1. P. betulaefolia CNGCs Possess Conserved Family Structures

Cyclic nucleotide-gated ion channels (CNGCs) are tetrameric cation channels that govern plant Na^+^, K^+^, and Ca^2+^ uptake, and their biological roles have been well documented in numerous herbaceous species [9,32]. Nevertheless, functional investigations of the CNGC family remain scarce for fruit-tree rootstocks, which are critical for stress adaptation in orchards. In the present work, we identified 26 *PbCNGC* members from *P. betulaefolia*, a stress-tolerant wild pear species widely used as rootstock for pear cultivation. Phylogenetic analysis classified these *PbCNGC* proteins into five established subgroups (Figure 1), consistent with the classification reported across various plant lineages [23,30,32,36,37,38,39,40,41,42,43].

Considerable variation in CNGC copy number exists among plant genomes. Genomic comparison between wild *P. betulaefolia* and domesticated *P. bretschneideri* revealed five *PbCNGC* paralogs unique to the wild *P. betulaefolia*, distributed in subgroups I, II, III, and IV-A, respectively. Such copy-number divergence may arise from long-term evolutionary adaptation in wild *P. betulaefolia*, coupled with gene loss events occurring during the domestication of cultivated *P. bretschneideri*. It is reasonable to speculate that these retained extra paralogs could contribute to the superior stress-adaptive capacity of wild *P. betulaefolia* rootstock, though their individual biological functions remain to be further verified.

Conserved-motif distribution and multiple-sequence alignment further supported the phylogenetic clustering of *PbCNGCs*. Motifs 1, 3, 7, 12, and 16 were universally preserved across all identified CNGC proteins, indicating their fundamental importance for CNGC biochemical activity [27,29,41]. In addition, the canonical conserved amino-acid signature [LI]-X(2)-[GS]-X-[FYIVS]-X-G-X(0,1)-[DE]-LL-X(8,25)-[SA]-X(9)-[VLIT]-E-X-F-X-[IL], where X stands for any amino acid residue and the numerals in parentheses represent the quantity of intervening amino acids, within the CNBD domain was detected in *PbCNGC* proteins. The phosphate-binding cassette and hinge region inside the CNBD are indispensable for CNGC activation. The preservation of these canonical structural features implies that *PbCNGC4* and *PbCNGC14*, the two salt-responsive members characterized in this study, likely follow the general activation mode of plant *CNGCs*. Meanwhile, variations in non-core motifs among subgroups may underlie functional divergence, such as differences in ion-selectivity or stress-response specificity among different *PbCNGC* paralogs [23,32,39,40,41,42,43].

### 3.2. PbCNGCs Mediate the Salt Stress Response in P. betulaefolia

Accumulating evidence confirms that cyclic nucleotide-gated ion channels (CNGCs) exert vital functions in plant responses to biotic and abiotic stresses [4,9]. In particular, CNGCs are generally involved in plant adaptation to salt stress, and their functions are relatively conserved across plant species [27,33]. Our analyses revealed that *PbCNGCs* possess the conserved structural features typical of plant *CNGC* families and undergo transcriptional changes under NaCl treatment (Figure 5 and Figure 6). Moreover, CNGCs from diverse plant species usually confer salt tolerance by maintaining Ca^2+^ homeostasis via hormone signaling pathways [38]. Here, sequence analysis showed that the promoters of *PbCNGC* genes contain abundant cis-regulatory elements associated with stress and hormone responses (Figure 3). Even so, whether hormone signals dominate the transcriptional regulation of *PbCNGCs* remains to be verified by further experiments.

Quantitative expression analysis indicated that six *PbCNGC* members exhibited a typical expression profile of initial upregulation followed by downregulation under salt stress, with transcript abundance peaking at different time points after NaCl treatment. For both genotypes, the expression peaks of these genes appeared earlier in roots than in stems and leaves (Figure 7). Specifically, *PbCNGC3*, *PbCNGC4*, and *PbCNGC14* displayed differential expression across roots, stems, and leaves, respectively. *PbCNGC10* showed leaf-preferential expression, whereas *PbCNGC19* and *PbCNGC20;1* were mainly differentially expressed in roots. Collectively, the divergent tissue-specific expression patterns and dynamic salt-responsive transcriptional characteristics of *PbCNGCs* drive functional specialization of the *CNGC* family in *P. betulaefolia*, thereby improving the salt adaptability of this rootstock.

Plant CNGCs act as core cation channels mediating K^+^ and Na^+^ transport. The high conservation of CNGC amino acid sequences across species implies conserved ion transport functions [22,23,26,27]. For example, *Arabidopsis AtCNGC1* and *AtCNGC4* exhibit equal permeability to K^+^ and Na^+^ [24,44]. In the present study, yeast complementation assays verified that *PbCNGC4* and *PbCNGC14* could effectively rescue impaired ion uptake and transport in yeast mutants and markedly facilitate transmembrane transport of Na^+^ and K^+^ (Figure 10). The transcription of these two *PbCNGC* genes shows spatiotemporal divergence, and such expression variations modulate the cation transport activity of PbCNGC4 and PbCNGC14 proteins. These findings uncover functional diversification within the *PbCNGC* family and provide a basis for dissecting the potential molecular mechanism governing salt tolerance in *P. betulaefolia*.

### 3.3. PbCNGC4 and PbCNGC14 Regulate Salt Tolerance via DNA Methylation

DNA methylation, particularly promoter region methylation, acts as a core epigenetic mechanism that regulates gene transcription and mediates plant salt adaptation under abiotic stress [11]. Previous studies have reported that salt stress-induced DNA demethylation can activate the expression of ion transport and stress-responsive genes, thereby maintaining cellular ion homeostasis and improving plant salt tolerance [5]. In this study, significant methylation differences in *PbCNGC4* and *PbCNGC14* were detected in the roots of two *P. betulaefolia* genotypes. Under salt stress, the salt-tolerant genotype exhibited a substantially greater reduction in methylation levels of these two genes in roots than the ordinary genotype, which led to a much higher increase in their transcriptional abundance in the salt-tolerant genotype and further resulted in differences in root Na^+^/K^+^ contents between the two genotypes [10]. These results indicate that, beyond spatiotemporal and tissue-specific transcriptional variations, epigenetic modifications of *PbCNGCs* play a pivotal role in shaping the salt-responsive expression patterns in ordinary (O) and salt-tolerant (T) *P. betulaefolia* genotypes. Exogenous bioactive compounds can improve crop salt tolerance [12,13,14,15]. For example, 5-azaC treatment reduces DNA methylation and activates stress-responsive genes in kenaf to boost salt tolerance [45]. Comparable responses occur in *P. betulaefolia*, where 5-azaC triggers DNA demethylation and K^+^ enrichment [10]. Accordingly, we hypothesize that the salt-tolerant genotype (T) keeps a “pre-activated” hypomethylated state at the *PbCNGC4* and *PbCNGC14* loci, enabling fast transcriptional reprogramming under salt stress. In contrast, the ordinary genotype (O) possesses a hyper-methylated epigenetic barrier that represses ion-transporter production, causing Na^+^ over-accumulation, K^+^ efflux, and salt sensitivity [10]. These genotype-dependent methylation variations explain transcriptional divergence and efficient ion homeostasis in salt-tolerant plants [10]. Thus, targeted modification of *CNGC* methylation may serve as a promising strategy for breeding salt-tolerant crops.

In cotton, demethylation of *GhCNGC4* mediated by the demethylase GhALKBH10B participates in drought responses by altering m^6^A methylation levels and modulating mRNA stability, confirming that methylation modification serves as a vital upstream regulatory mechanism for *CNGC* stress responses [34]. Most existing studies have centered on methylation-dependent regulation of CNGC genes in response to drought stress, whereas the epigenetic mechanisms governing CNGC-associated salt-stress responses remain poorly characterized. Combined with our transcriptomic data, the differential methylation levels of *PbCNGC4* and *PbCNGC14* between the two genotypes may largely account for their divergent spatiotemporal expression patterns under salt stress, ultimately driving the functional differentiation of *PbCNGC* family members.

CNGC-mediated Ca^2+^ influx acts as an upstream trigger of the salt-stress signaling cascade, promoting Na^+^ efflux and maintaining Na^+^/K^+^ homeostasis in plants. The resultant Ca^2+^ signal can be perceived by Ca^2+^-dependent protein kinase (CDPK), which functions as a Ca^2+^-binding Ca^2+^ sensor to execute downstream calcium signal transduction [9]. For instance, the synergistic regulation of GsCNGC20-d and GsCDPK29 contributes to salt-stress response and adaptation in the salt-tolerant wild soybean *Glycine soja* BB52 [27]. Our previous studies have verified that CDPK family genes, as core downstream components of Ca^2+^ signal transduction, can decode specific Ca^2+^ signatures and activate downstream stress-responsive pathways, playing an indispensable role in the salt tolerance regulation of *P. betulaefolia* [10]. In this study, yeast complementation assays further functionally verified the ion transport capacity of *PbCNGC4* and *PbCNGC14* proteins and clarified their specific functions in Na^+^/K^+^ transport. However, whether methylation modification affects the ion transport functions of *PbCNGC4* and *PbCNGC14* by regulating gene expression remains to be experimentally validated. Collectively, we speculate that methylation-mediated transcriptional regulation of *PbCNGC4* and *PbCNGC14* modulates the intensity and temporal rhythm of intracellular Ca^2+^ influx under salt stress, thereby precisely regulating the expression levels and functional activity of downstream *PbCDPK* genes. However, the specific interaction mode between *CNGCs* and downstream *CDPKs* remains unclear and requires further experimental verification. The epigenetic–transcriptional synergistic regulation model of *CNGCs* identified in this study accounts for the differential salt stress responses among different *P. betulaefolia* genotypes, offering a novel epigenetic perspective for further dissecting the functional differentiation mechanism of plant *CNGC* families and improving the regulatory network of salt stress responses.

## 4. Materials and Methods

### 4.1. Plant Materials and Treatments

The salt-tolerant (T, 6‰ NaCl-tolerant) and ordinary (O, 3‰ NaCl-tolerant) genotypes of *Pyrus betulaefolia* were maintained in the pear germplasm nursery of Jiangsu Academy of Agricultural Sciences [10]. Mature seeds were collected in the autumn of 2024. After stratification and germination treatment, the seeds were sown in a mixed substrate consisting of nutrient soil and vermiculite at a ratio of 3:1. All seedlings were cultivated in a greenhouse at 25 °C, with 70% relative humidity and a 16-h light photoperiod. Seedlings at 90 days after germination were carefully rinsed to remove root substrate and prepared for subsequent different treatments. Forty-eight seedlings of each genotype were transferred to Hoagland’s nutrient solution supplemented with 200 mM NaCl for quantitative real-time polymerase chain reaction (qPCR) analysis. The roots, stems, and leaves were harvested after being treated with 200 mM NaCl for 0, 1, 2, 4, 6, 8, 12, and 24 h (with three biological replicates) to detect target gene expression levels.

Twenty-four seedlings of each genotype were transferred to Hoagland solution for the methylation experiment. The DNA methylation inhibitor 5-azacytidine (5-azaC, Solarbio, Beijing, China) and accelerator methyl trifluoromethanesulfonate (MTFMS, TCI, Tokyo, Japan) were applied to regulate DNA methylation levels. 5-azaC + NaCl and MTFMS + NaCl groups were supplied with 100 μmol∙L^−1^ 5-azaC or 100 μmol∙L^−1^ MTFMS in Hoagland solution for 12 h. Meanwhile, the control and NaCl groups grew in Hoagland solution for 12 h. Then, the control group was provided with fresh Hoagland solution, and the other three groups were transferred to Hoagland solution supplemented with 200 mmol∙L^−1^ NaCl for 4 h. Finally, roots from four groups were collected for qPCR and McrBC-qPCR (McrBC digestion coupled quantitative real-time PCR) analysis, respectively.

### 4.2. Identification and Analysis of CNGC Genes

Similar to previous research [46], protein sequences of *Arabidopsis thaliana* CNGCs were used as queries for BLASTP (v2.14.0) searches (E-value < 1 × 10^−5^) against the *P. betulaefolia* genome deposited in the NCBI database to identify CNGC homologs (accessed on 8 March 2025). Meanwhile, the Hidden Markov Model (HMM) profiles of the ion transport (PF00520) and CNBD (PF00027), obtained from the Pfam database (https://www.ebi.ac.uk/interpro/, accessed on 10 March 2025), were used to search for CNGC proteins [29]. The results from both methods were merged, and redundant sequences were removed. Then, these protein sequences were submitted to the Conserved Domains Database (https://www.ncbi.nlm.nih.gov/Structure/cdd/cdd.shtml, accessed on 10 March 2025), SMART database (http://smart.embl-heidelberg.de/, accessed on 10 March 2025), and Pfam database (https://pfam.xfam.org/, accessed on 10 March 2025) for domain analysis [30]. Finally, the physicochemical properties, including isoelectric points (pI), amino acid compositions, and molecular weight, were calculated using the “Protein Parameter Calc” function in the TBtools-II software (v2.376) [47]. Subcellular localization of CNGC proteins was predicted using the WoLF PSORT online server (https://wolfpsort.hgc.jp/, accessed on 11 March 2025) [48].

These genes were aligned with 20 *AtCNGC* [36] and 21 *PbrCNGC* [37] sequences using the MUSCLE program in the TBtools-II software (v2.376) [47]. Then, a neighbor-joining phylogenetic tree was constructed with 1000 bootstrap replicates, and its visualization optimization was conducted on EvolView (https://www.evolgenius.info/evolview/, accessed on 11 March 2025). In addition, other feature analyses and visualizations of the CNGC gene family—including motif characterization, gene structure analysis, promoter element analysis, chromosomal distribution mapping, and FPKM heatmap—were also performed using the TBtools-II software (v2.376) [47]. The DESeq2 software package (v1.46.0) in R (v4.4.1) was employed to identify differentially expressed genes (DEGs) in the transcriptomes of two genotypes before and after salt stress (NCBI, project number PRJNA812627), with the screening criteria of fold change ≥2 and false discovery rate (FDR) < 0.05 [10]. Subsequently, specific CNGC members among these DEGs were screened based on their gene IDs (Table 1), and a Venn diagram was generated using the EVenn online tool (https://www.bic.ac.cn/test/venn/#/, accessed on 12 March 2025).

### 4.3. Methylation Analysis and Differentially Methylated CNGC Genes

Genome-wide methylation data of *P. betulaefolia* was downloaded from NCBI (project number PRJNA812739) to analyze the methylation status of this species, and methylation analysis was carried out as described previously [10]. Then, Bedtools (v2.21.0) [49] in R package (v4.4.1) was utilized to annotate the genetic characteristics of all differentially methylated regions (DMRs). Differentially methylated genes (DMGs) were defined as the set of genes that overlap between DMRs and DEGs identified from the transcriptome data. Ultimately, the differentially methylated *CNGC* family members were identified based on their gene IDs (Table 1).

### 4.4. qPCR and McrBC-qPCR

RNA extraction and cDNA synthesis were performed as previously described [10]. In the present study, the Primer3 online tool (http://primer3.ut.ee, accessed on 15 March 2025) was employed to design qPCR primers specific to six *CNGC* genes from *P. betulaefolia* (Table 3). After validating primer specificity, the qPCR reaction mixture was prepared in strict compliance with the protocol of Genious 2 × SYBR Green Fast qPCR Mix (ABclonal, Wuhan, China), with the thermal cycling program set as follows: an initial denaturation step at 95 °C for 30 s, followed by 40 cycles of denaturation at 95 °C for 5 s and annealing/extension at 60 °C for 30 s. Here, the internal control gene was *PbEF-1α* (GWHTAAYT007379) and the expression levels of the *CNGC* genes relative to *PbEF-1α* were determined via the 2^−ΔΔCT^ algorithm [10]. Genomic DNA (gDNA) isolation and enzymatic digestion with the restriction endonuclease McrBC (New England Biolabs, Beverly, USA) were performed as previously described [10]. The design and specificity verification for McrBC-qPCR primers were consistent with the above qPCR assays (Table 3). Notably, the qPCR signal intensity is negatively correlated with the methylation level of the target region in McrBC-qPCR results [50].

### 4.5. Yeast Complementation Experiment

To assess the roles of *PbCNGC4* and *PbCNGC14* in Na^+^ or K^+^ uptake, *Saccharomyces cerevisiae* genotypes *B31* [22] and *CY162* [35] were used for yeast complementation assays, respectively. The full-length cDNAs of *PbCNGC4* or *PbCNGC14* were cloned into the yeast expression vector pYES2 (Invitrogen, Carlsbad, CA, USA) downstream of the *GAL1* promoter between the *KpnI* and *XbaI* restriction sites, and transformed into *B31* and *CY162* via MicroPulser electroporation (Bio-Rad, Hercules, CA, USA), respectively. Transformants of *B31* and *CY162* were selected on an SD basal medium supplemented with uracil dropout supplement (-Ura DO, Clontech, San Francisco, CA, USA).

Following 16 h of pre-cultivation of individual transformants in liquid SD-Ura medium at 30 °C, 1 mL aliquots of the yeast suspension were centrifuged at 5000× *g* for 1 min, and the supernatants were discarded. Yeast cells were rinsed three times with Na^+^-free AP medium and subsequently adjusted to an optical density at 600 nm (OD_600_) of 1.0. For the Na^+^ influx assay in *B31* transformants, 0, 50, or 100 mM NaCl was supplemented into the AP medium. All yeast genotypes were subjected to 1:10 serial dilution, after which 10 μL of each diluted suspension was spotted onto corresponding agar plates. The plates were then incubated at 30 °C for 72 h to allow cell growth. For growth rate evaluation, the washed cells were resuspended in liquid AP medium with 0, 50, or 100 mM NaCl to an OD_600_ of 0.01 and incubated at 30 °C for 48 h. Then, aliquots were collected for OD_600_ determination. For K^+^ influx assay in *CY162* transformants, all procedures were performed as described above, except that potassium-free modified YNB medium supplemented with 2, 50, or 100 mM KCl was used for yeast cultivation.

### 4.6. Data Analysis

All experiments were performed with three independent biological replicates, and each biological replicate included three technical replicates. Statistical analyses were conducted using Origin 2025 software (https://www.originlab.com/). One-way analysis of variance (ANOVA) combined with Tukey’s honestly significant difference test was used for pairwise comparisons of *PbCNGC* gene expression and yeast complementation data, and different lowercase letters indicate significant differences at *p* < 0.05. Two-sample *t*-test combined with hypothesis testing was used for comparisons of methylation levels and gene expression levels between the two *P. betulaefolia* genotypes in methylation experiments, and asterisks (*) indicate significant differences at *p* < 0.05. All data were expressed as mean ± standard error (SE) with three biological replicates.

## 5. Conclusions

*P. betulaefolia* harbors 26 conserved *CNGC* family members, which respond to salt stress via distinct spatiotemporal expression patterns. *PbCNGC4* and *PbCNGC14* could restore Na^+^ and K^+^ uptake capacity in mutant yeast, and their transcriptional levels are modulated by demethylation modification under salt stress. In salt-tolerant genotypes, salt stress induces a substantial reduction in the methylation levels of *PbCNGC4* and *PbCNGC14*, which significantly upregulates their gene expression and further affects the absorption and transport of sodium and potassium ions in roots.

## Figures and Tables

**Table 1:** Characteristics of 26 cyclic nucleotide-gated ion channel (*CNGC*) genes in *Pyrus betulaefolia*.

Gene Name	Group	Gene ID	Position	Subcellular Localization	Coding Sequence Length/bp	Protein Length/aa	Conserved Domains	Conserved Domains’ Location	Relative Molecular Weight/kDa	Theoretical Isoelectric Point (pI)
*PbCNGC1*	I	GWHGAAYT050302	GWHAAYT00000007: 26,403,195–26,406,463: +	Plasma membrane	2142	713	PLN03192 cNMP domain SM000100	626–636 IQAAWRRHMKK	82.43	9.13
*PbCNGC3*	I	GWHGAAYT002107	GWHAAYT00000001: 17,099,483–17,102,711: +	Plasma membrane	2142	713	PLN03192 cNMP domain SM000100	627–637 IQAAWRRHMKK	82.47	9.11
*PbCNGC10*	I	GWHGAAYT000814	GWHAAYT00000001: 8,365,044–8,368,150: +	Plasma membrane	2166	721	PF00520 cNMP domain SM000100	651–661 IQAAWRRHWKR	82.78	10.02
*PbCNGC11;1*	I	GWHGAAYT002146	GWHAAYT00000001: 17,375,383–17,378,488: −	Plasma membrane	1962	653	cNMP domain SM000100	No	75.31	9.71
*PbCNGC11;2*	I	GWHGAAYT053237	GWHAAYT00000008: 22,546,955–22,552,615: +	Plasma membrane	1815	604	cNMP domain SM000100	593–603 IQATWRRRHGR	68.94	9.92
*PbCNGC12*	I	GWHGAAYT002109	GWHAAYT00000001: 17,116,814–17,121,863: +	Plasma membrane	2107	668	cNMP domain SM000100	582–592 IQAAWRRHMKK	75.78	9.41
*PbCNGC13*	I	GWHGAAYT002147	GWHAAYT00000001: 17,393,165–17,396,179: −	Plasma membrane	1878	625	cNMP domain SM000100	No	71.76	9.64
*PbCNGC21*	I	GWHGAAYT002105	GWHAAYT00000001: 17,090,683–17,093,096: −	Plasma membrane	1782	593	cNMP domain SM000100	No	68.78	9.05
*PbCNGC5;1*	II	GWHGAAYT020856	GWHAAYT00000015: 9,290,815–9,294,361: +	Plasma membrane	2150	719	PLN03192 cNMP domain SM000100	633–643 IQAAWRRYSKR	81.91	9.74
*PbCNGC5;2*	II	GWHGAAYT040814	GWHAAYT00000005: 4,374,730–4,378,058: −	Plasma membrane	2223	740	PLN03192 cNMP domain SM000100	651–661 IQAAWRHYRRK	84.81	8.87
*PbCNGC6*	II	GWHGAAYT031139	GWHAAYT00000002: 497,679–503,220: +	Plasma membrane	2385	794	cNMP domain SM000100	708–718 IQAAWRRYSKR	90.22	9.76
*PbCNGC14*	III	GWHGAAYT020433	GWHAAYT00000015: 6,417,019–6,420,004: −	Plasma membrane	2241	746	PLN03192 cNMP domain SM000100	644–654 IQVAWRRFKKK	86.02	9.07
*PbCNGC15*	III	GWHGAAYT018774	GWHAAYT00000014: 21,111,829–21,114,953: −	Plasma membrane	2114	704	PLN03192 cNMP domain SM000100	588–598 IQVAWRRFRKR	80.97	8.91
*PbCNGC16;1*	III	GWHGAAYT007497	GWHAAYT00000011: 12,444,624–12,447,975: −	Plasma membrane	1986	661	PF00520 cNMP domain SM000100	587–588 IQAAWRRCKKR	75.73	8.82
*PbCNGC16;2*	III	GWHGAAYT035281	GWHAAYT00000003: 9,498,373–9,501,909: −	Plasma membrane	2103	700	PF00520 cNMP domain SM000100	622–632 IQAAWRRSKKR	80.38	9.27
*PbCNGC17*	III	GWHGAAYT027204	GWHAAYT00000016: 21,786,091–21,791,824: +	Plasma membrane	2079	692	PLN03192 cNMP domain SM000100	598–608 IQAAWRRHKRR	79.36	8.92
*PbCNGC18*	III	GWHGAAYT053695	GWHAAYT00000009: 2,038,273–2,041,602: −	Plasma membrane	1815	604	PF00520 cNMP domain SM000100	488–498 IQAAWRRFKKR	69.25	8.6
*PbCNGC19*	IV A	GWHGAAYT047144	GWHAAYT00000006: 23,953,978–23,969,075: +	Plasma membrane	2391	796	PF00520 cNMP domain SM000100	754–755 IQVAWRYRKKC	89.72	8.52
*PbCNGC20;1*	IV A	GWHGAAYT019425	GWHAAYT00000014: 25,088,326–25,093,798: +	Plasma membrane	2334	777	PF00520 cNMP domain SM000100	741–751 IQVAWRYRKKC	88.61	9.57
*PbCNGC20;2*	IV A	GWHGAAYT047142	GWHAAYT00000006: 23,940,600–23,946,060: +	Plasma membrane	2424	807	PF00520 cNMP domain SM000100	769–779 IQVAWRYRKKC	91.73	8.65
*PbCNGC20;3*	IV A	GWHGAAYT054207	GWHAAYT00000009: 5,307,449–5,312,469: −	Plasma membrane	2040	679	PF00520 cNMP domain SM000100	647–657 IQVAWRYRKKR	77.74	9.32
*PbCNGC2*	IV B	GWHGAAYT028356	GWHAAYT00000017: 3,414,080–3,417,326: −	Plasma membrane	2076	691	PLN03192 cNMP domain SM000100	666–676 IQFAWRRYRLR	79.14	9.89
*PbCNGC4*	IV B	GWHGAAYT036687	GWHAAYT00000003: 24,919,487–24,926,754: −	Plasma membrane	2076	691	PLN03192 cNMP domain SM000100	635–645 IQLAWRRYKHR	79.78	8.35
*PbCNGC7*	IV B	GWHGAAYT053948	GWHAAYT00000009: 3,551,511–3,555,613: −	Plasma membrane	2139	712	PLN03192 cNMP domain SM000100	666–676 IQFAWRRYRLR	81.63	10.05
*PbCNGC8*	IV B	GWHGAAYT049912	GWHAAYT00000007: 24,009,828–24,013,592: +	Plasma membrane	2100	699	cNMP domain SM000100	656–666 IQLAWRRHRMR	80.75	9.56
*PbCNGC9*	IV B	GWHGAAYT009007	GWHAAYT00000011: 28,712,407–28,719,652: −	Plasma membrane	2076	691	PLN03192 cNMP domain SM000100	635–645 IQLAWRRYKHR	79.87	8.93

Note: (+) and (−) represent the forward and reverse orientations of genes on chromosomes, respectively.

**Table 2:** Differential methylation profiles of *PbCNGC* genes in roots of *Pyrus betulaefolia*.

Gene Name	The Type of Methylation	Gene ID	Chromosome	Methylation Start	Methylation End	Width	Methylation Differential	Significant	Annotation	Gene Start	Gene End	Gene Length	Distance to TSS
*PbCNGC4*	TR vs. TNR CHH	GWHGAAYT036687	03	24,922,001	24,922,200	200	−18.13	Hypo	Intron (2 of 6)	24,919,029	24,926,754	7726	4554
	TR vs. OR CHH	GWHGAAYT036687	03	24,919,001	24,919,200	200	10.07	Hyper	3′ UTR	24,919,029	24,926,754	7726	7554
*PbCNGC14*	TR vs. OR CHH	GWHGAAYT020433	15	6,421,801	6,422,000	200	−27.72	Hypo	Promoter (1–2 kb)	6,416,855	6,420,128	3274	−1673
	OR vs. ONR CHH	GWHGAAYT020433	15	6,421,201	6,421,400	200	13.27	Hyper	Promoter (1–2 kb)	6,416,855	6,420,128	3274	−1073
	OR vs. ONR CHH	GWHGAAYT020433	15	6,421,601	6,421,800	200	12.01	Hyper	Promoter (1–2 kb)	6,416,855	6,420,128	3274	−1473
	OR vs. ONR CHH	GWHGAAYT020433	15	6,421,801	6,422,000	200	28.45	Hyper	Promoter (1–2 kb)	6,416,855	6,420,128	3274	−1673

Note: OR and ONR denote root samples of the ordinary genotype collected before and after 24 h of 200 mM NaCl treatment, respectively. TR and TNR represent root samples of the salt-tolerant genotype prior to and following the same 200 mM NaCl treatment for 24 h. TSS refers to the transcription start site.

**Table 3:** Primer sequences of qPCR and McrBC-qPCR in this study.

Gene Name	Gene ID	Forward Primer (5′→3′)	Reverse Primer (5′→3′)	Target Amplicon Size/bp	Destination
*PbCNGC3*	GWHGAAYT002107	CCTGCCCGATACAAGATGAAGA	AATAGCACCAACCCAGCGAT	229	qPCR
*PbCNGC4*	GWHGAAYT036687	GCAACGGGGCTCATCATAGA	AGACCTTGCACTAGTGTCGC	243	qPCR
		AGGTCCAACTTTTTGCCCTCA	CAACCGTGATGCAGGTGATTG	232	McrBC-qPCR
*PbCNGC10*	GWHGAAYT000814	AGGCTGGTGACTTCTGTGGA	CGAAACTGGGAGGCGACAAA	160	qPCR
*PbCNGC14*	GWHGAAYT020433	GTGCGAGCGTTTGGTATCCT	GAGGGTTGATTTGGGAAGCA	217	qPCR
		ATGTTCGAAAGACAGGCGGA	TGGGATTGTTCATGCCACCA	244	McrBC-qPCR
*PbCNGC19*	GWHGAAYT047144	TAGGTTTCTGCCTCTGCTCG	TCATTTCCACGCCCACAATC	228	qPCR
*PbCNGC20;1*	GWHGAAYT019425	AGCCATAGACGCTTACCAGA	GGTTCATCCATCAGGGCAAA	191	qPCR

Note: qPCR, quantitative real-time polymerase chain reaction. McrBC-qPCR, McrBC digestion coupled quantitative real-time PCR.

## Data Availability

Data will be made available on request.
